# Supplementary material for: Stable MOB1 interaction with Hippo/MST is not essential for development and tissue growth control
Source: Nat Commun. 2017 Sep 25;8:695. doi: 10.1038/s41467-017-00795-y (PMC5612953; doi:10.1038/s41467-017-00795-y)
Supplement: Supplementary file 1 — Supplementary Information [file 41467_2017_795_MOESM1_ESM.pdf]

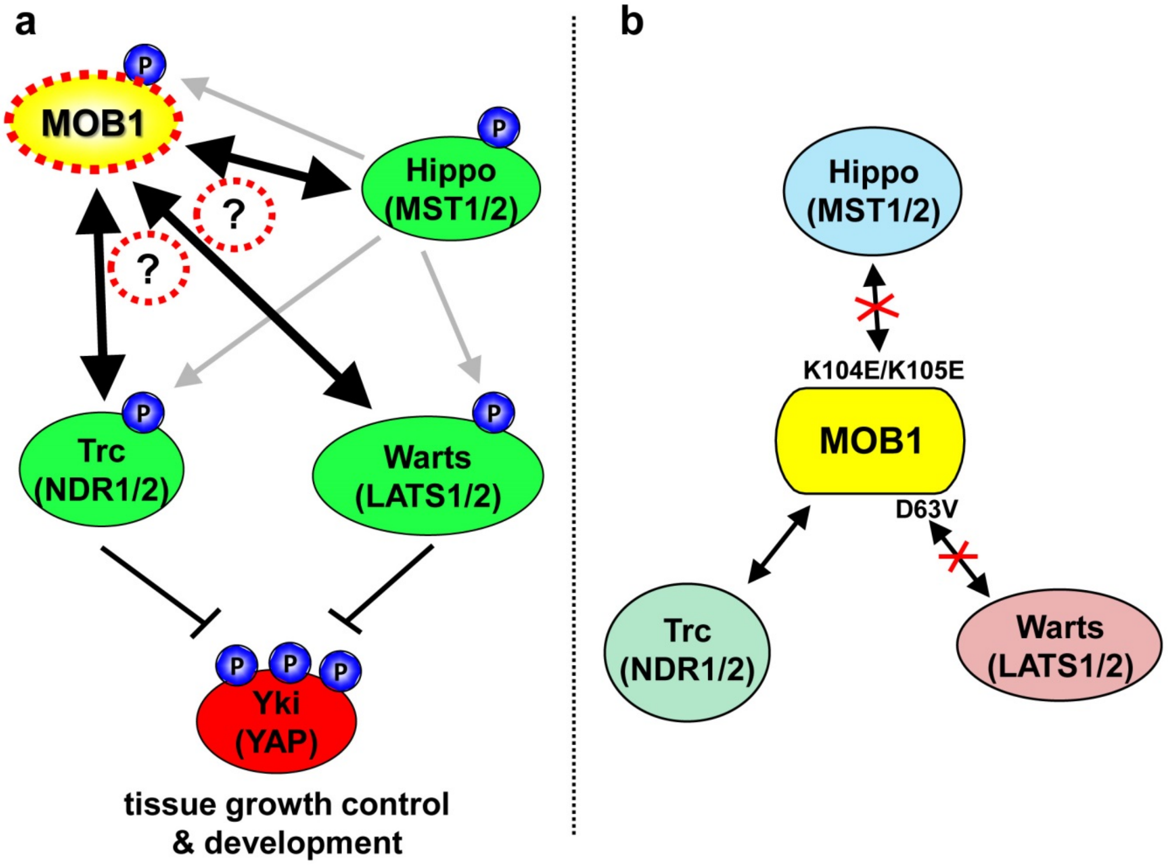

Supplementary Figure 1.

Key question and mutants of our study to decipher: How important are the regulatory interactions of MOB1 with Hippo core cassette kinases?

(a) Model illustrating key research questions regarding MOB1 as a central hub of Hippo core signaling: (1) Does MOB1 interact differently with MST1/2 (Hpo), LATS1/2 (Wts) and NDR1/2 (Trc) in human and fly cells? (2) If yes, is this influenced by MST1/2 (Hpo) mediated phosphorylation of MOB1? (3) Which interactions of MOB1 with MST1/2 (Hpo), LATS1/2 (Wts), or NDR1/2 (Trc) are biologically important in the context of Yki(YAP)-Hippo signaling, tissue growth control and development? **(b)** Schematic model of how the differential binding of MOB1 to MST1/2 (Hpo), LATS1/2 (Wts) and NDR1/2 (Trc) can be experimentally exploited using the D63V and K104E/K105E mutations in order to study the biological importance of these regulatory interactions of MOB1 with Hippo core kinases.

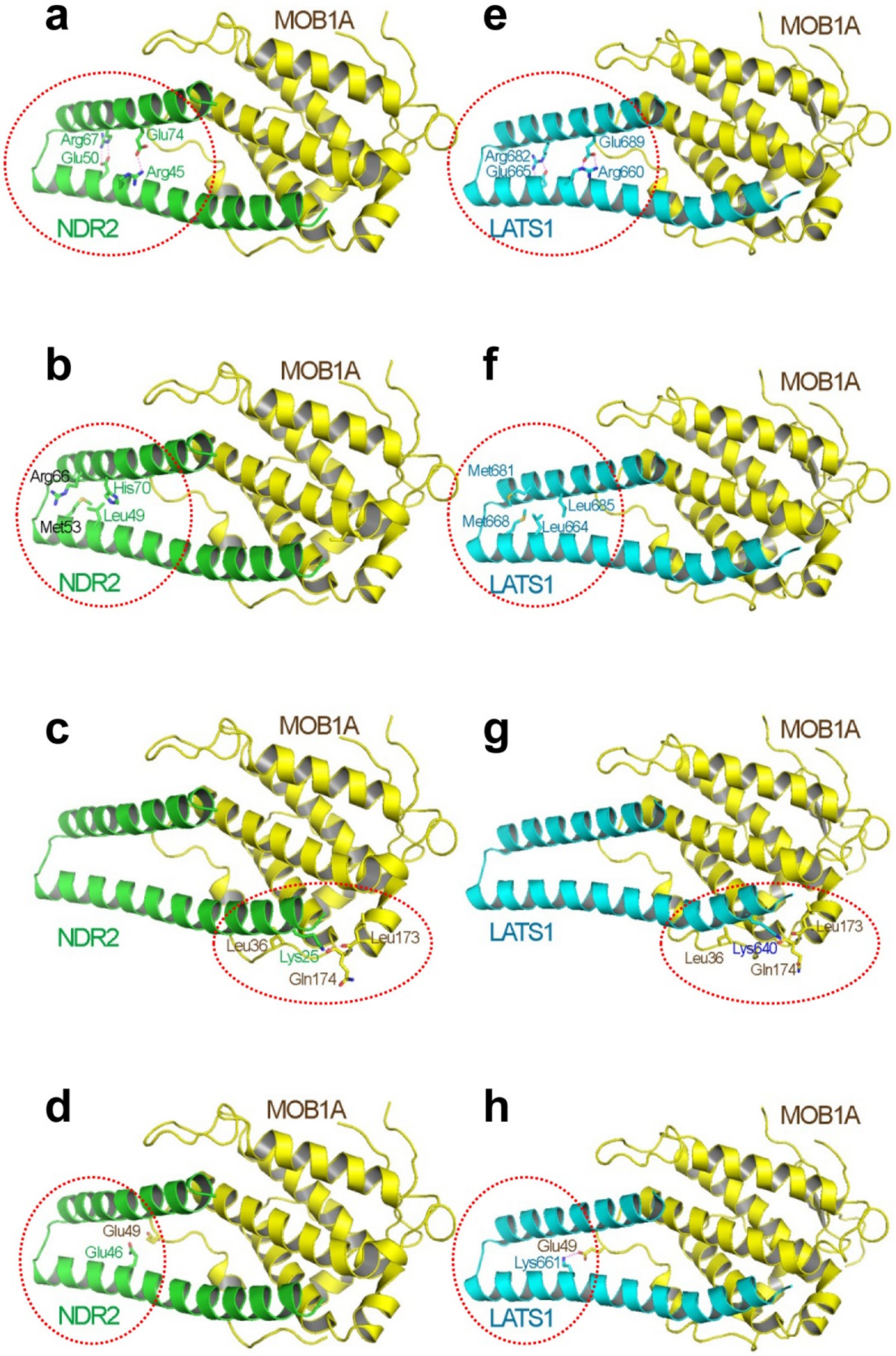

Supplementary Figure 2.

Structural comparison of MOB1 bound to the NTR domains of NDR2 and LATS1 (in support of Fig. 2).

To define whether MOB1 binding to NDR2 differs from binding to LATS1, we compared the published MOB1/LATS1 structures[19, 20] with our MOB1A/NDR2 structure (Fig. 1). For

comparison MOB1/NDR2 **(a-d)** and MOB1/LATS1 **(e-h)** complexes are shown with the compared areas highlighted by red circles. **(a,e)** Two conserved intramolecular interactions stabilize the V-shaped NTR domains of NDR2 (LATS1) mediated by Arg45 (Arg660), Glu50 (Glu665), Arg67 (Arg682), and Glu74 (Glu689). **(b,f)** The NTR of LATS1 forms additional stabilizing intramolecular bonds, involving Leu664, Met668, Met681 and Leu685; interactions that are not observed in the NTR of NDR2 (the corresponding residues are Leu49, Met53, Arg66 and His70 in NDR2). **(c,g)** In the first interaction interface Leu36, Leu173 and Gln174 of MOB1A interact with Lys25 of NDR2 (the corresponding residue is Lys640 in LATS1). Lys640 of LATS1, although not fully defined in the electron density map[19, 20], may interact with Gln174 and weakly with Leu36 and Leu173. However, biochemical data[63] suggest that the corresponding residue in NDR1, namely Lys24, is not essential for complex formation with MOB1. **(d,h)** Glu49 of MOB1A bonds with Lys661 of LATS1[19, 20], an interaction which is not observed between Glu49 of MOB1A and Gln46 of NDR2, the residue corresponding to Lys661 of LATS1. Additional notes: In the first interaction interface (see Fig. 1e) Gly39, Leu41, Ala44, Gln67, Met70, Leu71, and His185 of MOB1A display conserved intermolecular interactions with Leu28 (Met643), Tyr32 (Val647), Leu35 (Val650), Ile36 (Leu651) of NDR2 (LATS1). In the second interaction interface (see Fig. 1f) Glu51, Glu55, Phe132, Pro133, Ly135 and Val138 of MOB1A have conserved intermolecular interactions with Arg42 (Arg657), Thr75 (Ser690), Arg79 (Arg694) and Arg82 (Arg697) of NDR2 (LATS1). Moreover, Leu78 (Ile693) of NDR2 (LATS1) bonds with Trp56 and Val59 of MOB1A.

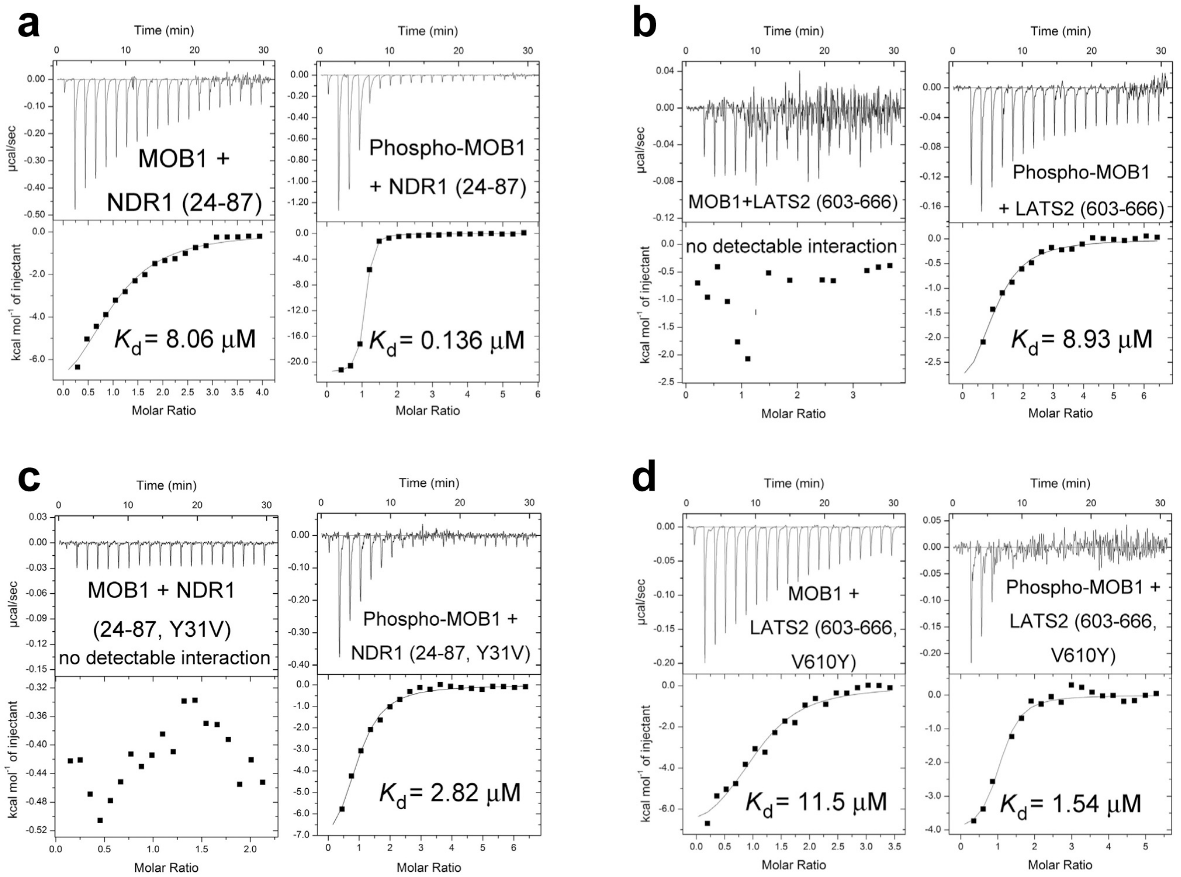

Supplementary Figure 3.

MOB1 binds differently to the NTR domains of NDR1 and LATS2 through key residues (in support of Fig. 2).

Isothermal titration calorimetry (ITC) assays measuring the dissociation constant (K_d) of indicated non-phosphorylated full-length MOB1 or MST2-phosphorylated MOB1 (phospho-MOB1) with wild-type and mutant NDR1 (24-87) or LATS2 (603-666) variants. (**a**) MOB1/NDR1 complex formation was dramatically increased by prior phosphorylation of MOB1. (**b**) An interaction between LATS2 wild-type and non-phosphorylated MOB1 was not detectable, while phospho-MOB1 bound to LATS2. (**c**) NDR1(Y31V) did not associate with non-phosphorylated MOB1, but bound to phospho-MOB1. (**d**) The mutation of Val610 to Tyr of LATS2 enabled LATS2 to bind to non-phosphorylated MOB1, and LATS2(V610Y) displayed an increased binding affinity for phospho-MOB1 compared to wild-type LATS2.

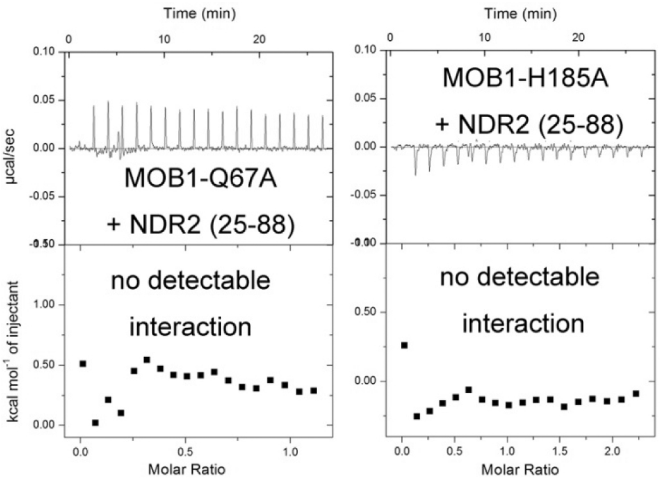

Supplementary Figure 4.

Characterization of the binding of MOB1 mutants with substitutions at Gln67 and His185 to the NTR of NDR2 (in support of Fig. 2).

Isothermal titration calorimetry (ITC) assays measuring the dissociation constant (K_d) of indicated non-phosphorylated full-length MOB1A mutants with wild-type NDR2 (25-88). Noteworthy, ITC measurements could not detect any interaction between NDR2 and MOB1(Q67A) or MOB1(H185A).

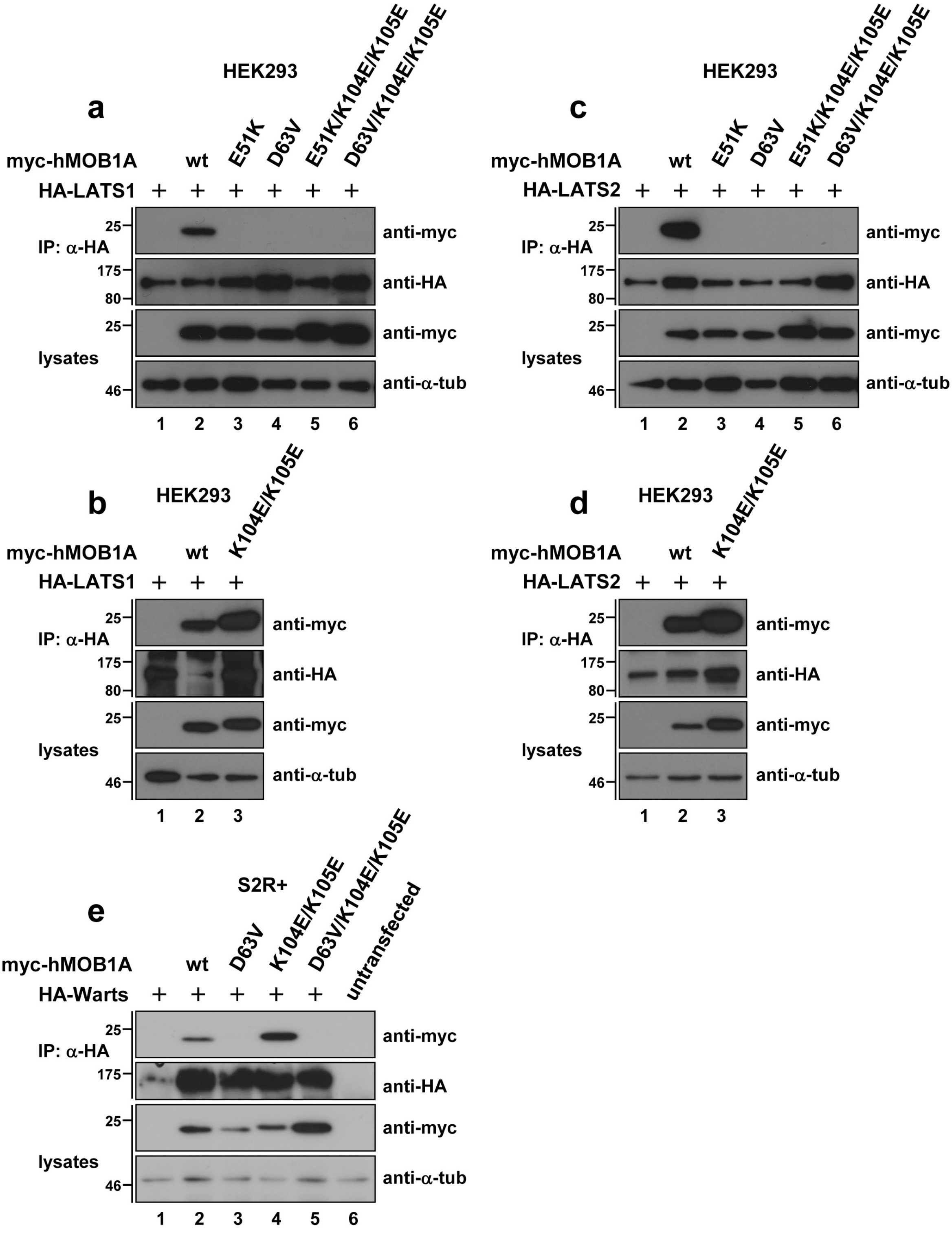

Supplementary Figure 5.

Characterization of the interaction of MOB1 variants with LATS1/2 and Warts kinases (in support of Fig. 3).

(a-d) Lysates of HEK293 cells expressing full-length HA-tagged LATS1 or LATS2 wild-type (wt) together with the indicated full-length MOB1A versions were subjected to immunoprecipitation (IP) using anti-HA 12CA5 antibody. Complexes were analyzed by immunoblotting using anti-myc (top) and anti-HA antibody (top middle). Input lysates were analyzed with anti-myc (bottom middle) and anti-α-tubulin antibody (bottom). Relative molecular weights are shown. The E51K and D63V mutations abolished MOB1 binding to LATS1/2, while the K104E/K105E mutant bound normally. **(e)** Lysates of *Drosophila* S2R+ cells expressing full-length HA-Wts(wt) together with indicated full-length MOB1A versions were subjected to immunoprecipitation (IP) using anti-HA. Complexes were analyzed by immunoblotting using anti-myc (top) and anti-HA (top middle). Input lysates were analyzed with anti-myc (bottom middle) and anti-α-tubulin (bottom). Relative molecular weights are shown. The D63V mutant did not bind to Warts, while the K104E/K105E mutant bound normally.

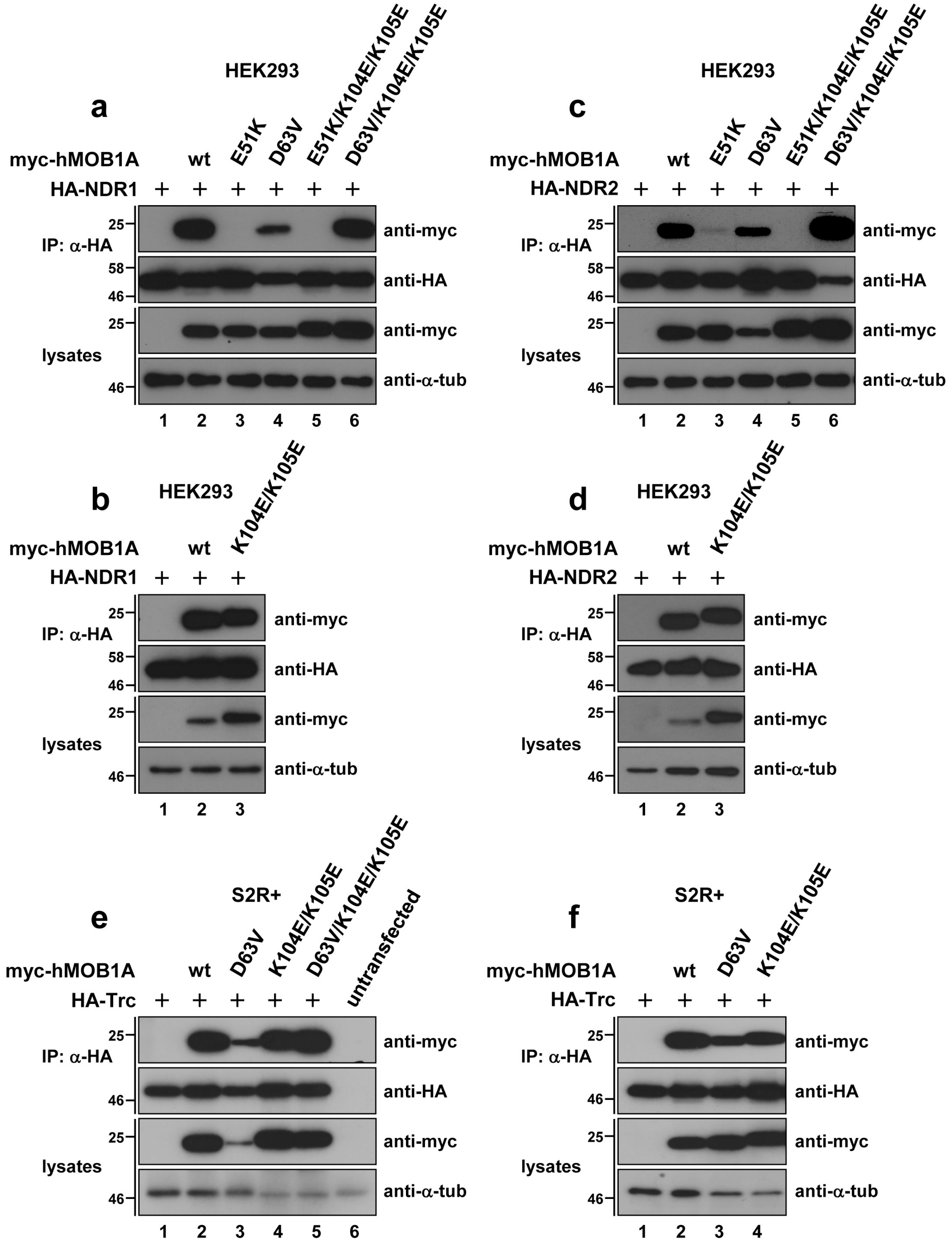

Supplementary Figure 6.

Characterization of the interaction of MOB1 variants with NDR1/2 and Trc kinases (in support of Fig. 3).

(a-d) Lysates of HEK293 cells expressing full-length HA-NDR1 or HA-NDR2 wild-type (wt) together with the indicated full-length MOB1A versions were subjected to immunoprecipitation (IP) using anti-HA 12CA5 antibody. Complexes were studied by immunoblotting using anti-myc (top) and anti-HA antibody (top middle). Input lysates were analyzed with anti-myc (bottom middle) and anti-α-tubulin antibody (bottom). Relative molecular weights are shown. Only versions carrying the E51K mutation displayed a reduction of binding to NDR1/2. **(e,f)** Lysates of *Drosophila* S2R+ cells expressing full-length HA-Trc(wt) together with indicated full-length MOB1A versions were subjected to immunoprecipitation (IP) using anti-HA. Complexes were examined by immunoblotting using anti-myc (top) and anti-HA (top middle). Input lysates were investigated with anti-myc (bottom middle) and anti-α-tubulin (bottom). Relative molecular weights are shown.

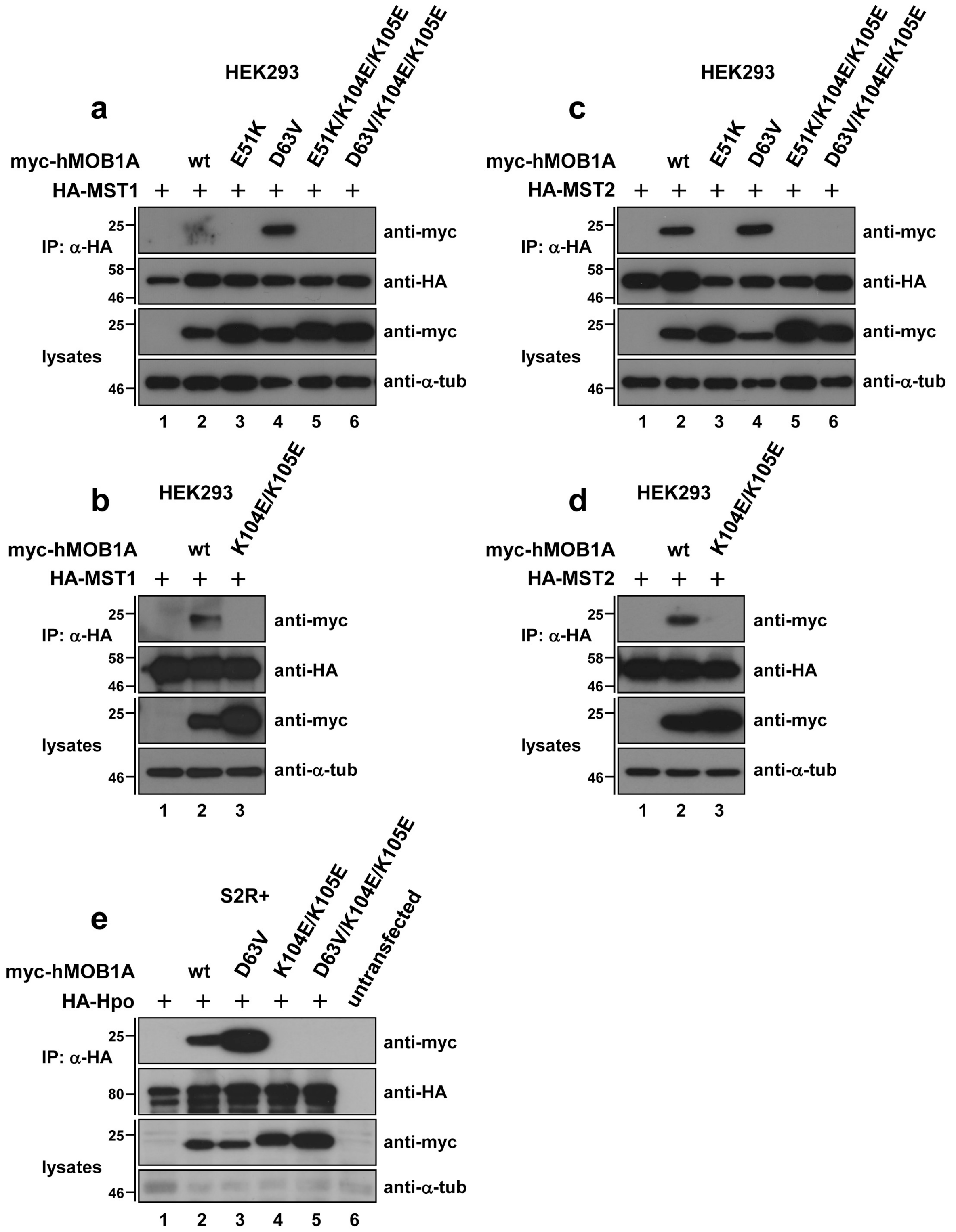

Supplementary Figure 7.

Characterization of the interaction of MOB1 variants with MST1/2 and Hpo kinases (in support of Fig. 3).

(a-d) Lysates of HEK293 cells expressing full-length HA-MST1 or HA-MST2 wild-type (wt) together with indicated full-length MOB1A versions were subjected to immunoprecipitation (IP) using anti-HA. Complexes were studied by immunoblotting using anti-myc (top) and anti-HA (top middle). Input lysates were analyzed with anti-myc (bottom middle) and anti-α-tubulin (bottom). Relative molecular weights are shown. The E51K and K104E/K105E mutations caused loss of binding to MST1/2, while the D63V mutant bound normally. **(e)** Lysates of *Drosophila* S2R+ cells expressing full-length HA-Hpo(wt) together with indicated full-length MOB1A versions were subjected to immunoprecipitation (IP) using anti-HA. Complexes were studied by immunoblotting using anti-myc (top) and anti-HA (top middle). Input lysates were probed with anti-myc (bottom middle) and anti-α-tubulin (bottom). Relative molecular weights are shown.

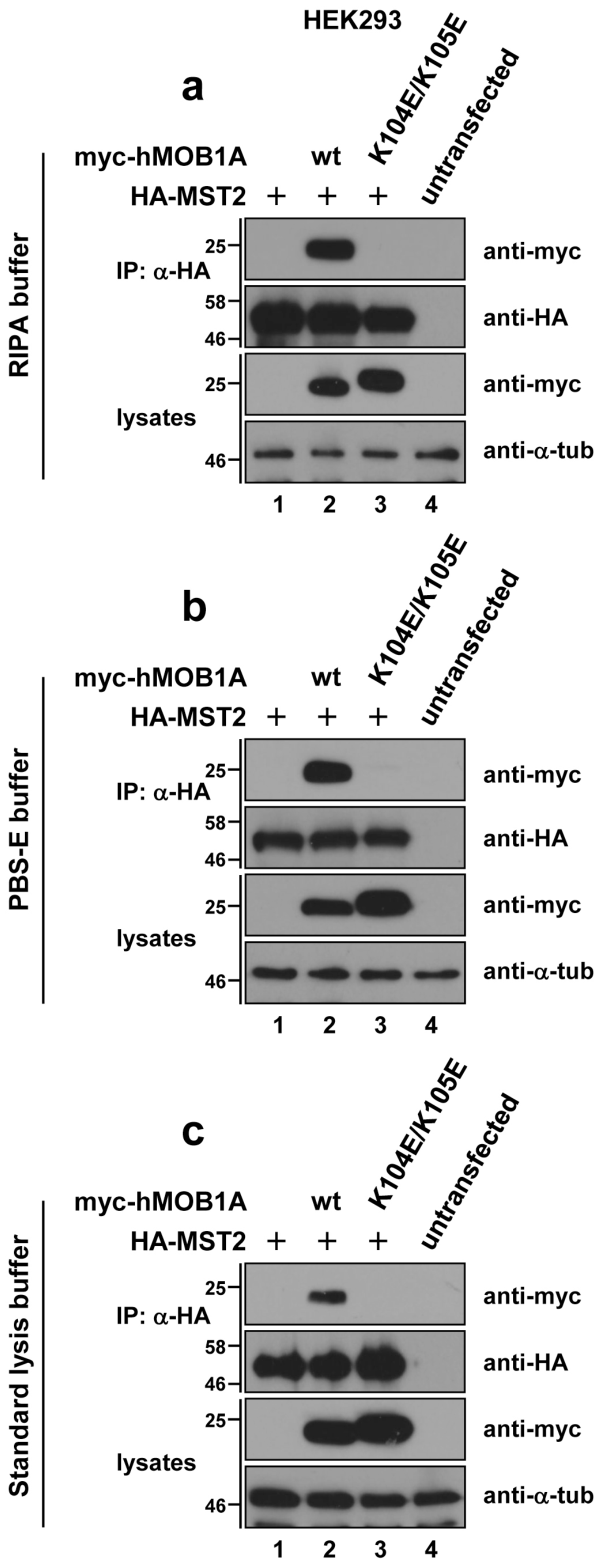

Supplementary Figure 8.

Characterization of MOB1(K104E/K105E) binding to MST2 in alternative lysis buffers (in support of Fig. 3).

Lysates of HEK293 cells expressing full-length HA-MST2 wild-type (wt) together with indicated full-length MOB1A versions were subjected to immunoprecipitation (IP) using anti-HA. Complexes were studied by immunoblotting using anti-myc (top) and anti-HA (top middle). Input lysates were analyzed with anti-myc (bottom middle) and anti-α-tubulin (bottom). Relative molecular weights are shown. The K104E/K105E mutations caused loss of stable MOB1 binding to MST2 irrespective of the lysis buffer conditions.

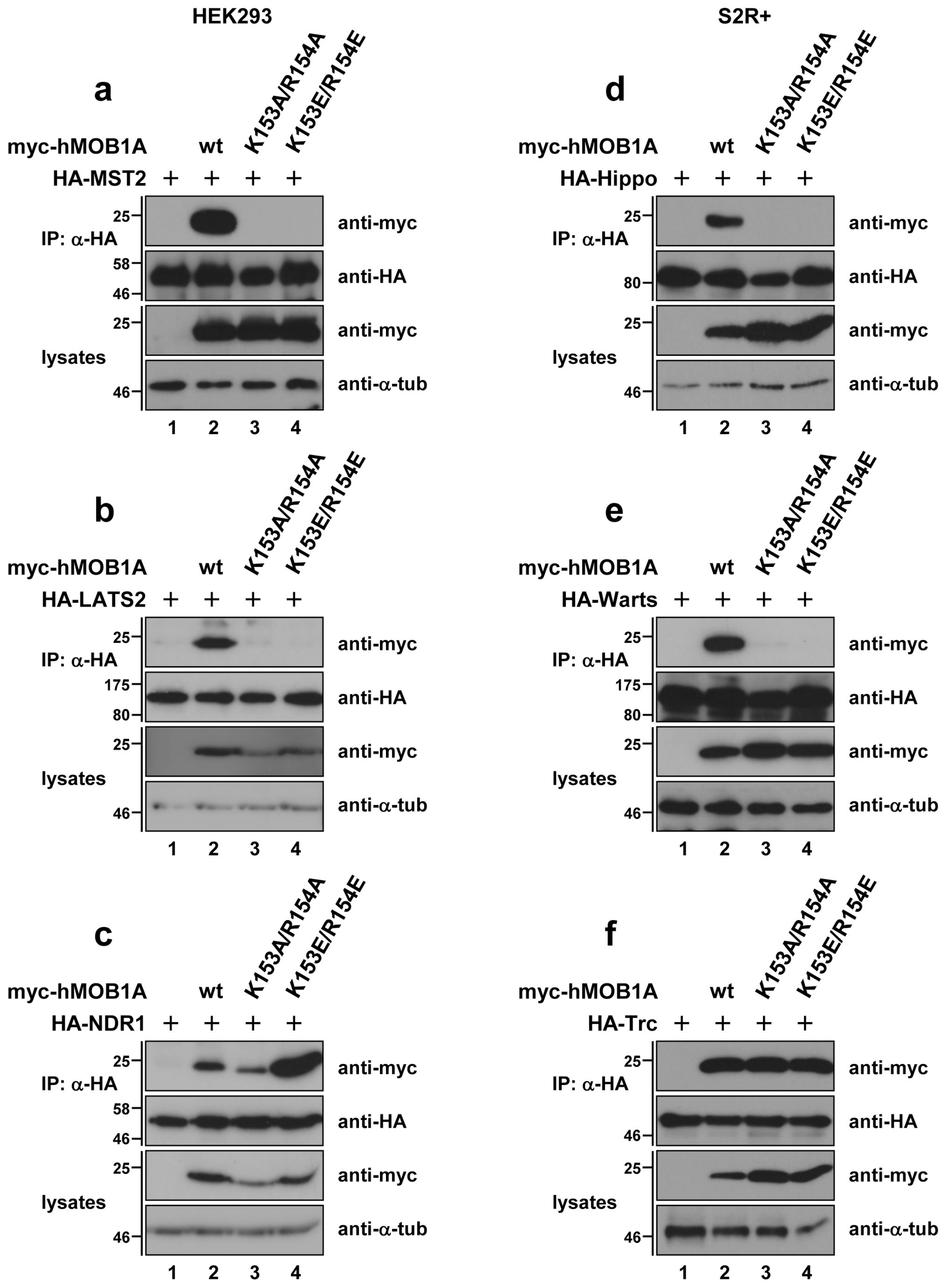

Supplementary Figure 9.

Characterization of MOB1(K153A/R154A) and MOB1(K153E/R154E) binding to human and fly Hippo core kinases (in context of Fig. 3).

(a-c) Lysates of HEK293 cells expressing full-length HA-MST2 wild-type (wt) **(a)**, HA-LATS2(wt) **(b)** or HA-NDR1(wt) **(c)** together with indicated full-length MOB1A versions were subjected to immunoprecipitation (IP) using anti-HA. Complexes were studied by immunoblotting using anti-myc (top) and anti-HA (top middle). Input lysates were analyzed with anti-myc (bottom middle) and anti-α-tubulin (bottom). K153/R154 mutations caused loss of stable MOB1 binding to MST2 and LATS2. **(d-f)** Lysates of *Drosophila* S2R+ cells expressing full-length HA-Hpo(wt) **(d)**, HA-Wts(wt) **(e)** or HA-Trc(wt) **(f)** together with indicated full-length MOB1A versions were subjected to IP using anti-HA. Complexes were studied by immunoblotting using anti-myc (top) and anti-HA (top middle). Input lysates were probed with anti-myc (bottom middle) and anti-α-tubulin (bottom). Relative molecular weights are shown.

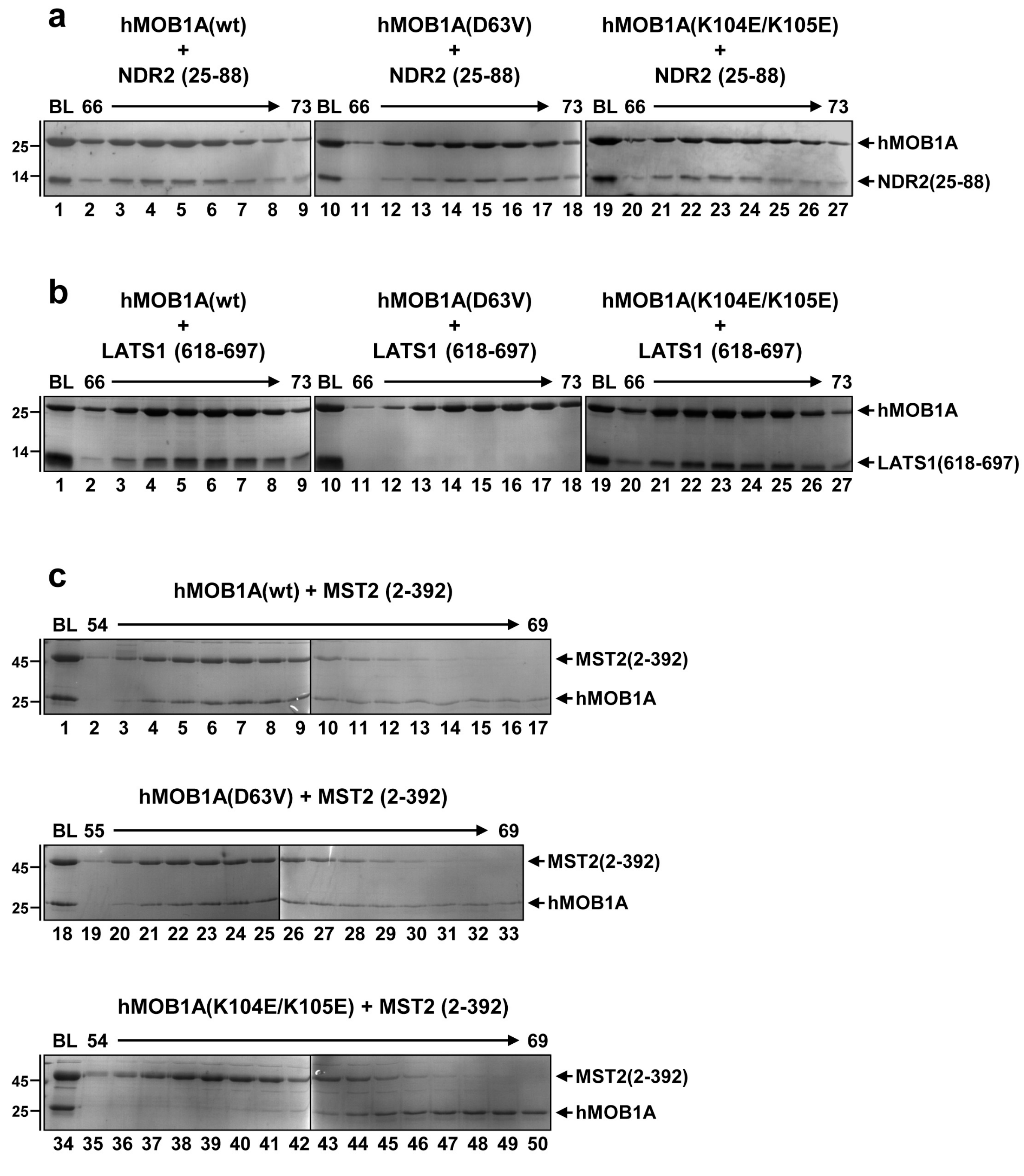

Supplementary Figure 10.

Characterization of MOB1(D63V) and MOB1(K104E/K105E) binding to NDR2, LATS1 and MST2 fragments by gel filtration chromatography (in support of Fig. 3).

(a-c) Indicated versions of recombinant MOB1A were incubated with recombinant wild-type NDR2(25-88), LATS1(618-697), or MST2(2-392), before loading (BL) onto a Superdex 200

column. To assess LATS1 binding, MST2 phosphorylated MOB1 proteins were used. The indicated fractions from the Superdex 200 column were analyzed by SDS-PAGE followed by Coomassie Blue staining. Relative molecular weights are shown. The positions of MOB1A, NDR2, LATS1 and MST2 are indicated on the right. (**a**) MOB1A(wt), MOB1A(D63V) and MOB1A(K104E/K105E) readily co-fractionated with NDR2. (**b**) Phosphorylated MOB1A(wt) and MOB1A(K104E/K105E) co-eluted with LATS1, while MOB1A(D63V) did not. (**c**) MOB1A(wt) and MOB1A(D63V) co-fractionated with MST2, but MOB1A(K104E/K105E) did not co-elute with MST2.

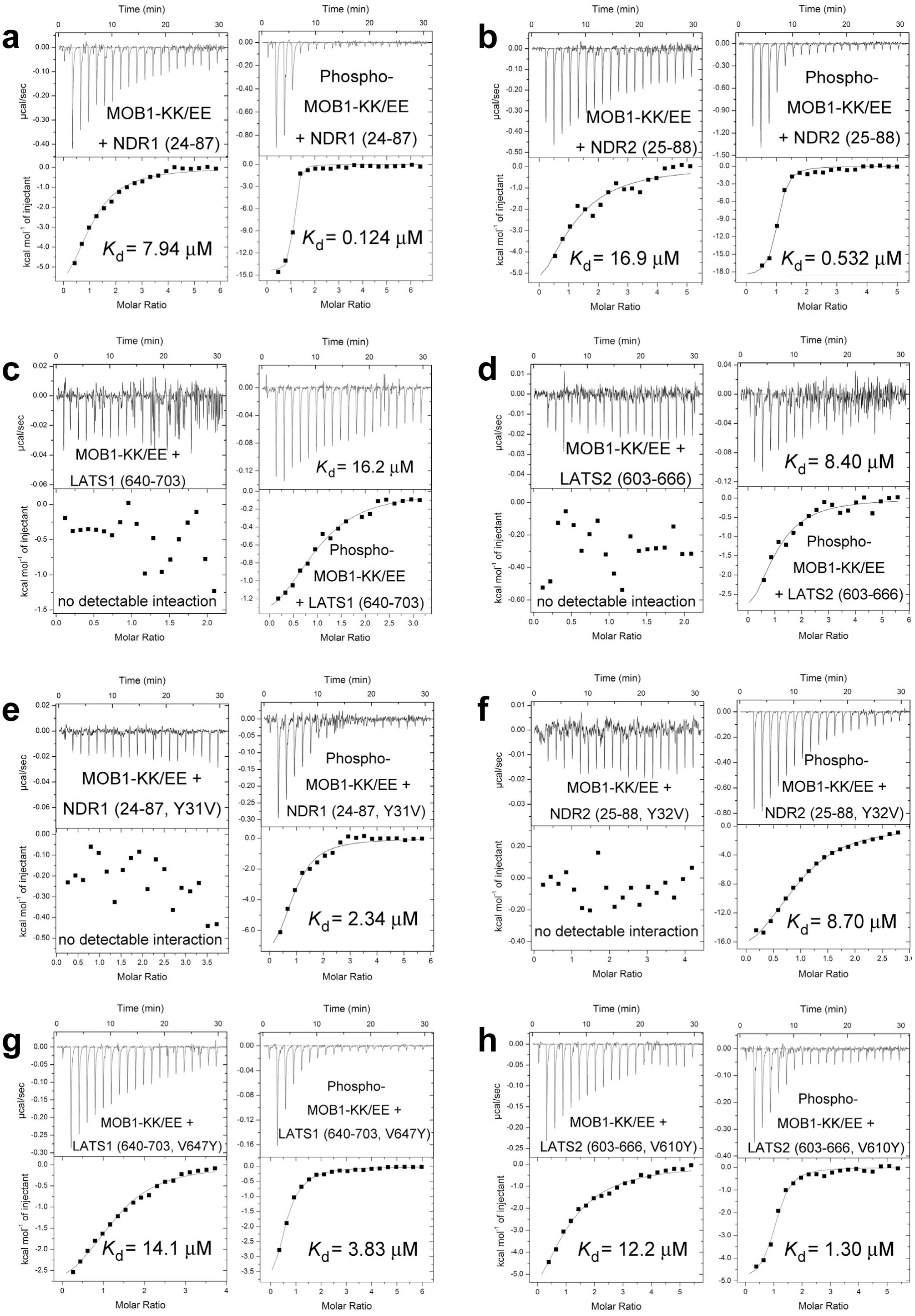

Supplementary Figure 11.

MOB1(K104E/K105E) interacts with the NTR domains of NDR1, NDR2, LATS1 and LATS2 similar to wild-type MOB1 (in support of Figs. 2 and 3).

ITC assays measuring the dissociation constant (K_d) of indicated non-phosphorylated full-length MOB1(K104E/K105E) or MST2-phosphorylated MOB1 (phospho-MOB1(K104E/K105E)) with wild-type and mutant NDR1 (24-87), NDR2 (25-88), LATS1 (640-703) or LATS2 (603-666) variants. Noteworthy, unphosphorylated and phospho-MOB1(K104E/K105E) displayed binding affinities to all four different NTRs that are very comparable to wild-type MOB1, suggesting that the K104E/K105E modifications do not alter MOB1 binding to the NTRs of NDR1, NDR2, LATS1 and LATS2 kinases.

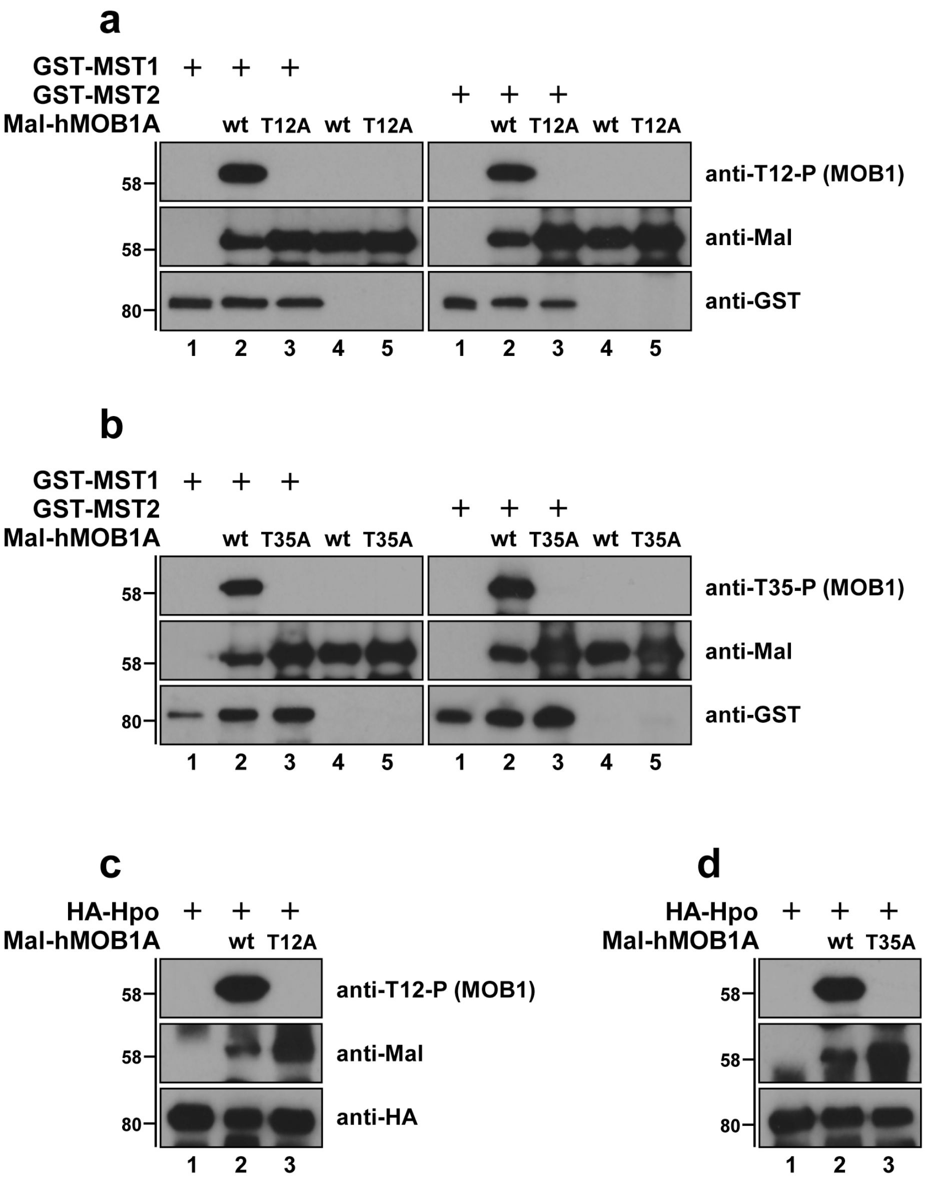

Supplementary Figure 12.

Characterization of the anti-Thr12-P and anti-Thr35-P antibodies in MST1/2(Hpo)-mediated phosphorylation of MOB1 (in support of Fig. 3).

(a,b) Recombinant full-length GST-MST1 or GST-MST2 wild-type (wt) were incubated with full-length recombinant Mal-MOB1A(wt) or the T12A and T35A phospho-acceptor mutants. Following kinase reactions, the samples were analyzed by Western blotting using indicated antibodies. Relative molecular weights are shown. Noteworthy, the anti-T12-P antibody only detected wild-type MOB1A when the kinase assays contained GST-MST1/2, while

MOB1A(T12A) was not detected (**a**). The anti-T35-P antibody also specifically detected the phosphorylation of MOB1A on Thr35 (**b**). **(c,d)** Lysates of *Drosophila* S2R+ cells transiently expressing full-length HA-Hpo(wt) were subjected to stringent immunoprecipitation with anti-HA 12CA5 antibody. Immunopurified proteins were then processed for kinase assays without or with the indicated full-length recombinant MOB1A versions. Following kinase reactions, the samples were examined by immunoblotting using the specified antibodies. Relative molecular weights are shown. Importantly, the anti-T12-P and anti-T35-P antibodies specifically detected Hpo-mediated phosphorylation of MOB1A on Thr12 (**c**) and Thr35 (**d**).

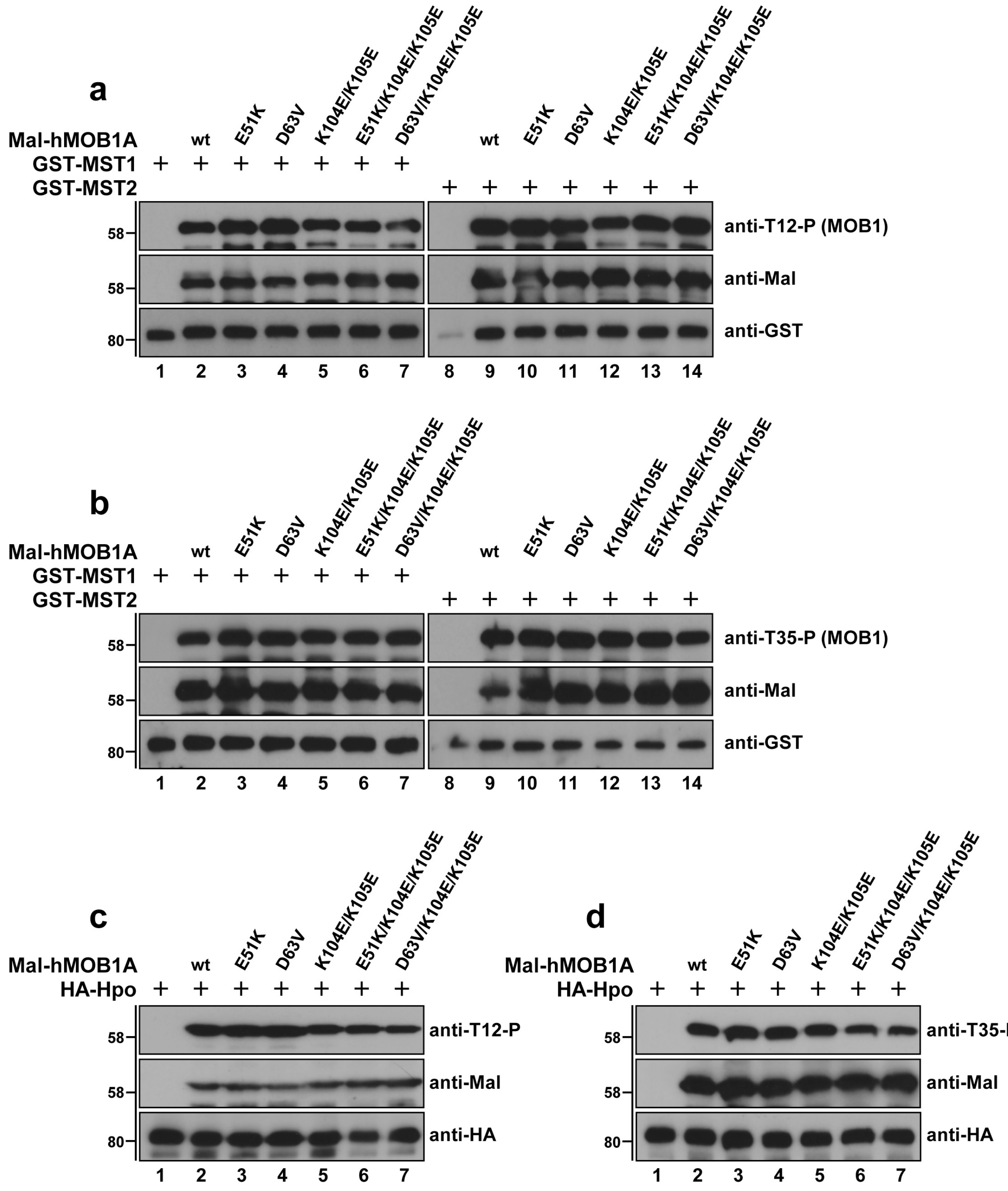

Supplementary Figure 13.

Characterization of the MST1/2(Hpo)-mediated phosphorylation of MOB1 variants (in support of Fig. 3).

(a,b) Recombinant full-length GST-MST1 or GST-MST2 wild-type (wt) were incubated with indicated full-length recombinant Mal-MOB1A variants. Following kinase reactions, the samples were analyzed by immunoblotting using the specified antibodies. Relative molecular

weights are indicated. **(c,d)** Lysates of *Drosophila* S2R+ cells transiently expressing full-length HA-Hpo(wt) were subjected to immunoprecipitation with anti-HA. Immunopurified Hpo was then processed for kinase assays with the indicated full-length recombinant MOB1A versions. Following kinase reactions, the samples were examined by immunoblotting using indicated antibodies. Relative molecular weights are shown.

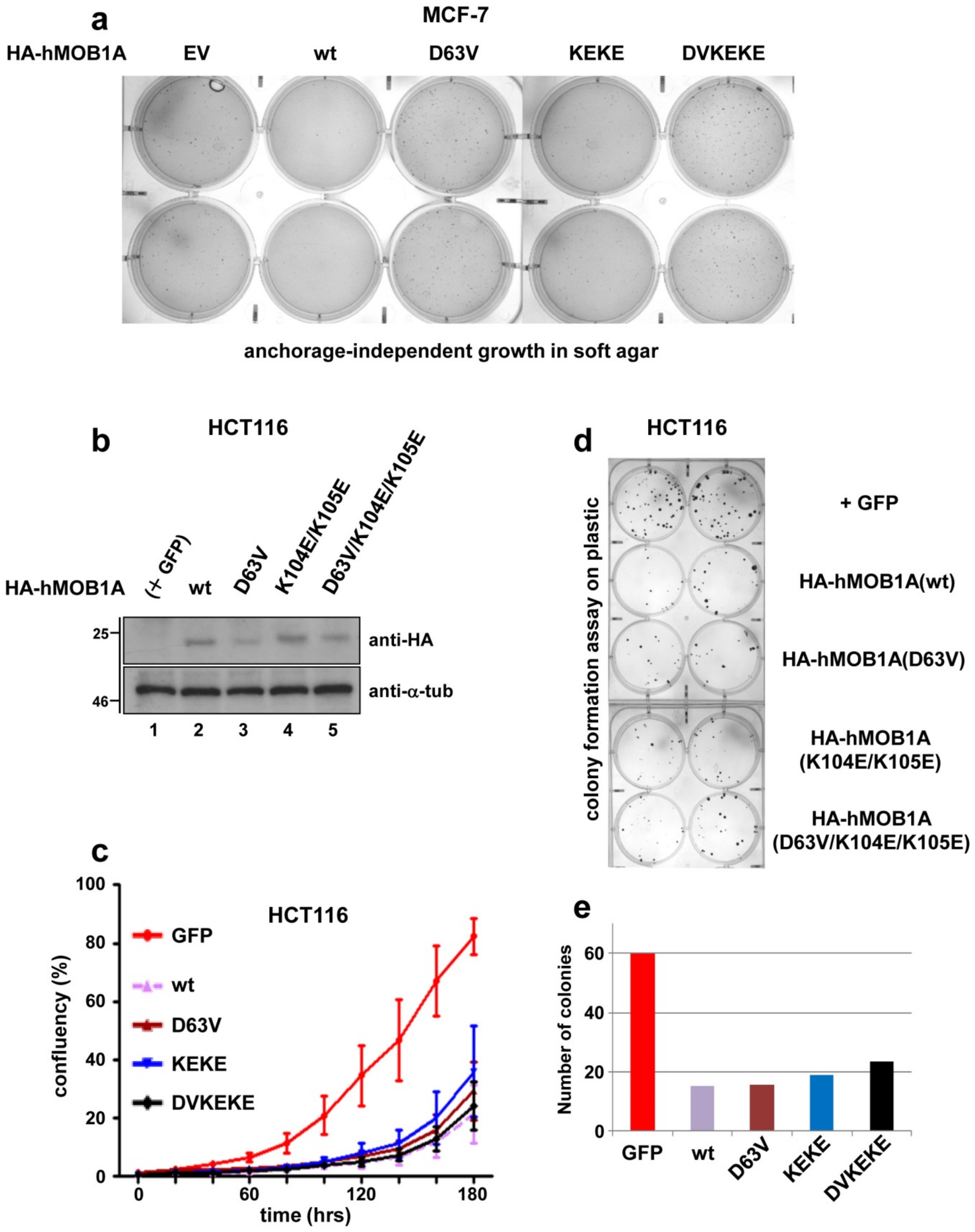

Supplementary Figure 14.

Anchorage-independent growth of MCF-7 cells, and cell proliferation and colony formation data for HCT116 colon cancer cells (in support of Fig. 4).

(a) Soft agar growth assays of the MCF-7 cells shown in Fig. 4e,f. Representative images are displayed. **(b)** Immunoblotting with indicated antibodies of cell lysates derived from HCT116 human colon cancer cells transiently expressing the indicated HA-MOB1 variants or GFP (+GFP) as negative control. Relative molecular weights are indicated. **(c)** Proliferation rates of attached HCT116 cells transiently expressing indicated HA-MOB1A variants. The average of two independent experiments performed in triplicates is shown. **(d)** Colony formation assays of the HCT116 cells shown in **b**. Representative images are displayed. **(e)** Quantifications of colony formation assays shown in **d**. The average of two experiments performed in duplicates is shown. wt, wild-type; DV, D63V; KEKE, K104E/K105E; DVKEKE, D63V/K104E/K105E.

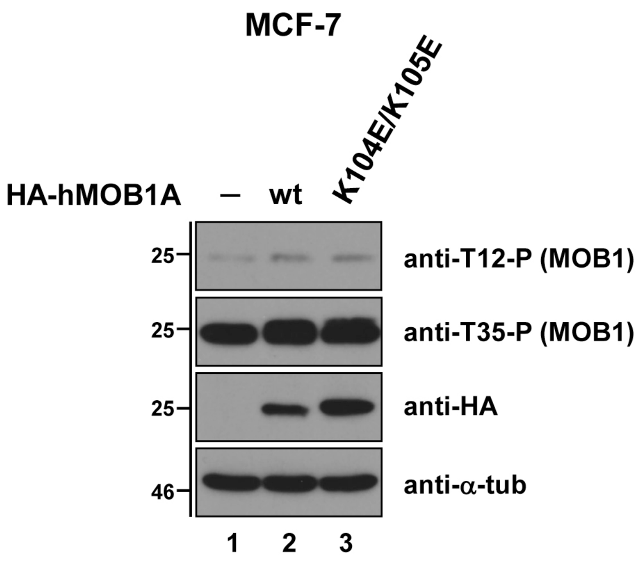

Supplementary Figure 15.

Characterization of the phosphorylation status of MOB1(K104E/K105E) in MCF-7 cells (in support of Figs. 3 and 4).

Immunoblotting with indicated antibodies of cell lysates derived from MCF-7 human breast cancer cells stably expressing the indicated HA-MOB1 variants or empty vector as negative control. Relative molecular weights are indicated. Noteworthy, Thr12 and Thr35 phosphorylation levels were comparable between HA-MOB1(K104E/K105E) and HA-MOB1 wild-type (wt), suggesting that loss of stable MST1/2 binding (due to the K104E/K105E modifications) does not impair MST1/2-mediated phosphorylation of MOB1 in MCF-7 cells.

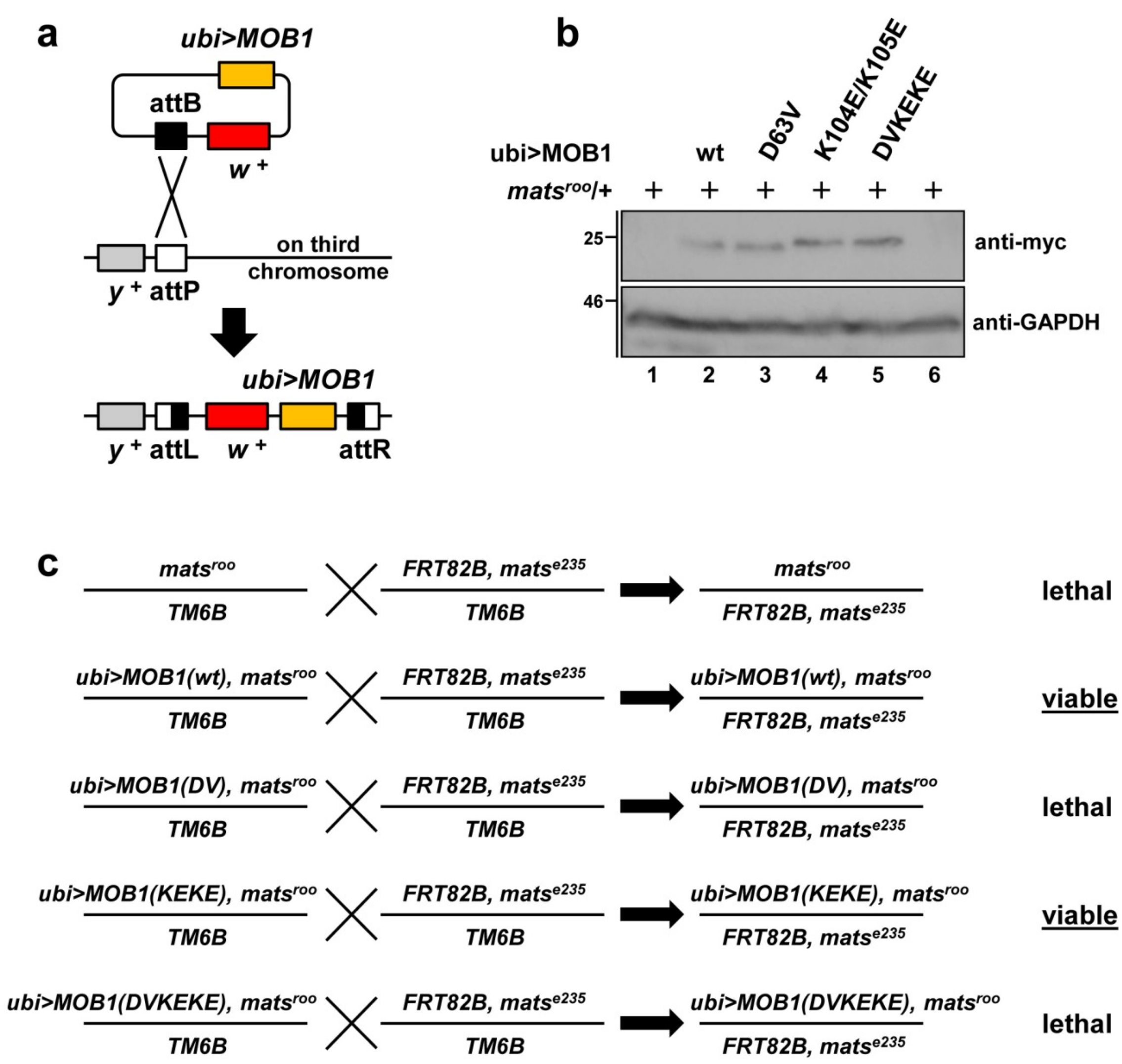

Supplementary Figure 16.

Ubiquitous expression of human MOB1(wt) or MOB1(K104E/K105E), but not MOB1(D63V) or MOB1(D63V/K104E/K105E), rescues larval lethality of *mats* mutant flies (in support of Figs. 5 and 6).

(a) Schematic representation of the generation of MOB1 transgenic flies. Using PhiC31-mediated recombination plasmids which can ubiquitously express N-terminally myc-tagged wild-type (wt) or mutant human MOB1 (ubi>MOB1) were inserted into one identical genome location in the same orientation (attP site at 89E11 on chromosome 3). **(b)** Whole flies of indicated genotypes were analyzed by Western blotting. Relative molecular weights are indicated. Similar expression of the myc-tagged MOB1 transgenes was observed. The

presence of the *mats*^{roo} allele was confirmed by PCR genotyping (data not shown). **(c)** Genetic schemes illustrating how we tested which MOB1 transgenes can rescue the larval lethality of Mats deficient flies. Adult flies expressing myc-MOB1(wt) or myc-MOB1(K104E/K105E) in a *mats* null (*mats*^{roo}/*mats*^{e235}) genetic background are viable and fertile, while adult flies expressing myc-MOB1(D63V) or myc-MOB1(D63V/K104E/K105E) were not observed. wt, wild-type; DV, D63V; KEKE, K104E/K105E; DVKEKE, D63V/K104E/K105E.

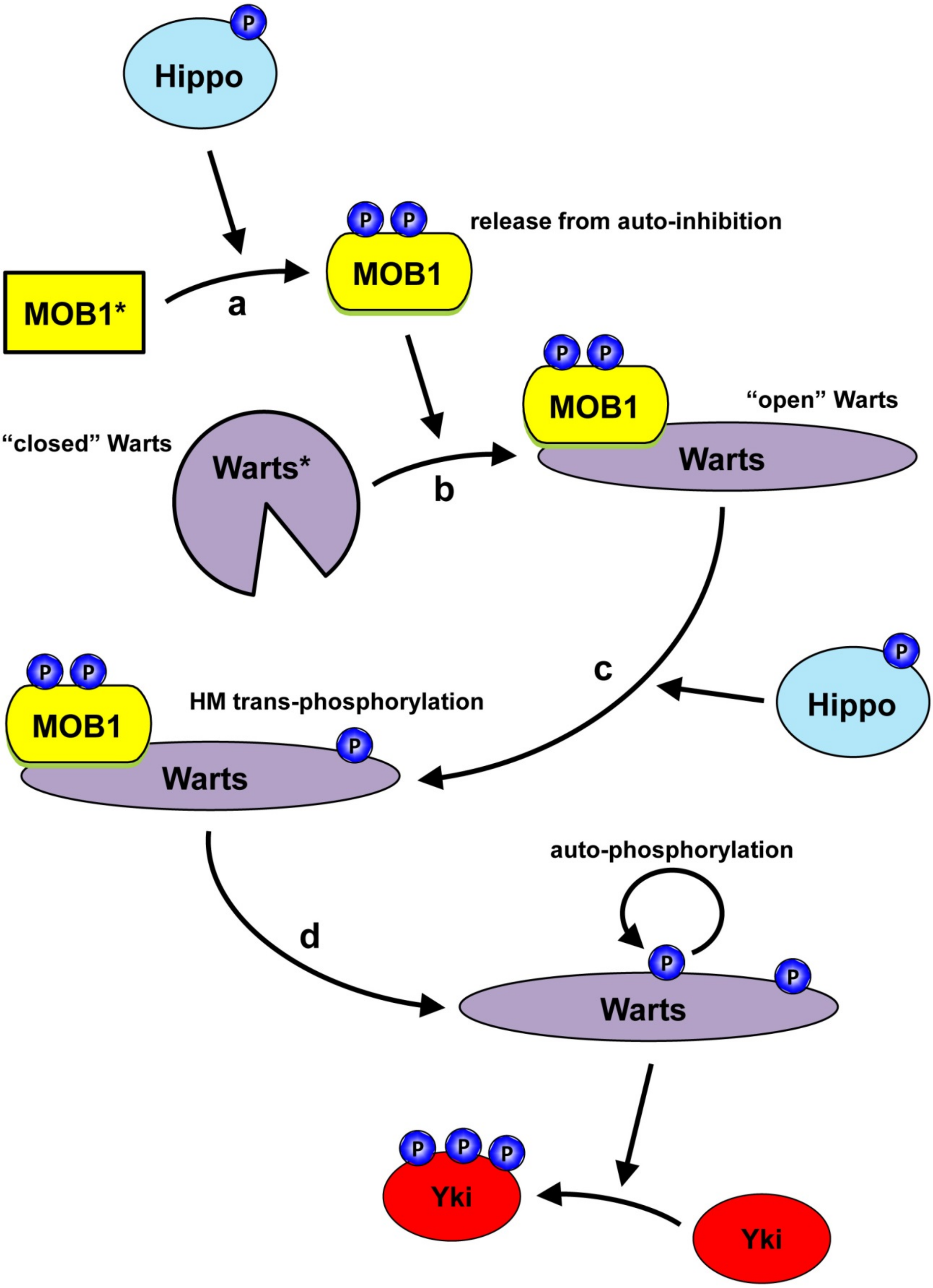

Supplementary Figure 17.

Proposed four-step model of the activation mechanism of MST1/2-MOB1-LATS1/2 signaling.

(a) Stimulated Hpo (MST1/2) phosphorylates MOB1, releasing MOB1 from its auto-inhibitory conformation. **(b)** MOB1 associates stably with Wts (LATS1/2), triggering a conformational change to "open up" Wts (LATS1/2). Noteworthy, although we indicate that Hpo (MST1/2) mediated phosphorylation of MOB1 plays an important role in this second step, we would like to stress that the importance of Hpo (MST1/2) phosphorylation is debatable for this second step (since according to biochemical data MOB1 phosphorylation by MST1/2 is required for MOB1 binding to LATS1/2 (see this study and refs.[19, 22]), while MOB1 activation of Wts can occur independently of Hpo in fly tissue[31]). **(c)** "Opened" Wts (LATS1/2) is phosphorylated by Hpo (MST1/2) in the C-terminal hydrophobic motif (HM) without the need to form a stable ternary MST1/2-MOB1-LATS1/2 complex. **(d)** HM-phosphorylated LATS1/2 can auto-phosphorylate itself in the central activation loop (T-loop), a step that can occur independent of MOB1 binding to LATS1/2. Finally, fully activated Wts (LATS1/2) can phosphorylate and thereby inhibit Yki (YAP). Taken together, we propose that Mats as well as Hpo act at least at two different steps in the sequential activation of Wts.

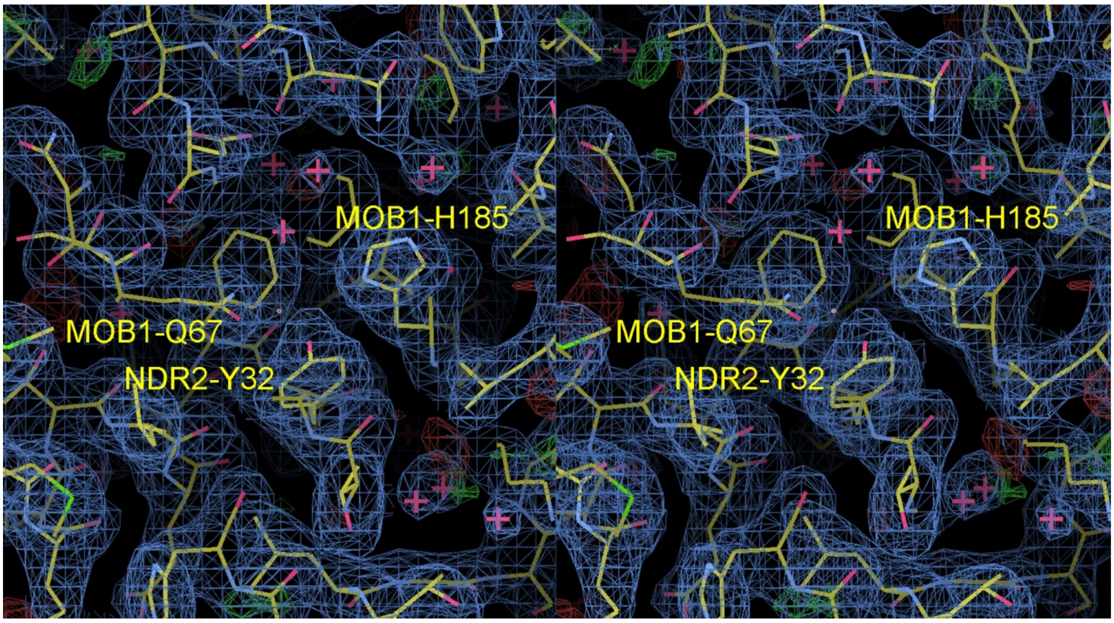

Supplementary Figure 18.

Stereo image of a portion of the electron density map of the MOB1/NDR2 complex.

The $2F_o{-}F_c$ electron density map at the MOB1-NDR2 interface is shown at the 1 σ contour level (cyan). The MOB1 and NDR2 structure models are superimposed. In the structure models, carbon, oxygen, and nitrogen atoms are colored in yellow, red, and blue, respectively. Water molecules are denoted as red crosses. The $F_o{-}F_c$ difference electron density map is displayed at the 2.5 σ contour level, with green and red representing positive and negative difference densities, respectively.

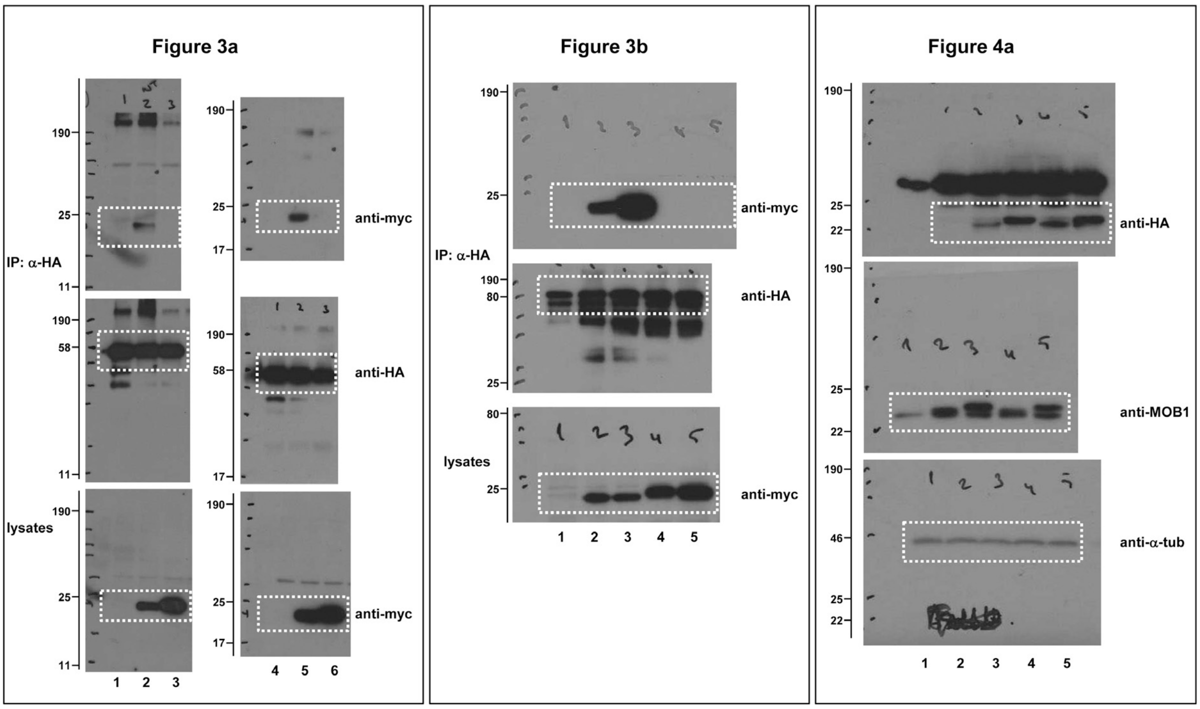

Supplementary Figure 19.

Uncropped Western blots for Figures 3a, 3b and 4a.

For details see the corresponding figure legends of Figures 3 and 4.
